# Quality of life among cancer inpatients 80 years and older: a systematic review

**DOI:** 10.1186/s12955-021-01685-0

**Published:** 2021-03-20

**Authors:** Jorunn Drageset, Reidun Karin Sandvik, Leslie Sofia Pareja Eide, Gunhild Austrheim, Mary Fox, Elisabeth Grov Beisland

**Affiliations:** 1 Faculty of Health and Social Sciences, Western Norway University of Applied Sciences, 5063 Bergen, Norway; 2 Department of Public Health and Primary Health Care, University of Bergen, Bergen, Norway; 3 Duke University, Toronto, Canada

**Keywords:** Quality of life, Instruments, 80 years and older, Cancer inpatient, Somatic hospitals, Systematic review

## Abstract

**Objective:**

The aim of this systematic review was to summarize and assess the literature on quality of life (QoL) among cancer patients 80 years and older admitted to hospitals and what QoL instruments have been used.

**Methods:**

We searched systematically in Medline, Embase and Cinahl. Eligibility criteria included studies with any design measuring QoL among cancer patients 80 years and older hospitalized for treatment (surgery, chemotherapy or radiation therapy). Exclusion criteria: studies not available in English, French, German or Spanish. We screened the titles and abstracts according to a predefined set of inclusion criteria. All the included studies were assessed according to the Critical Appraisal Skills Programme checklists, and the Preferred Reporting Items for Systematic Reviews and Meta-Analyses Statement checklist was used to ensure rigor in conducting and reporting. This systematic review was registered in PROSPERO (CRD42017058290).

**Results:**

We included 17 studies with 2005 participants with various cancer diagnoses and Classification of Malignant Tumors stages (TNM). The included studies used a range of different QoL instruments and had different aims and outcomes. Both cancer-specific and generic instruments were used. Only one of the 17 studies used an age-specific instrument. All the studies included patients 80 years and older in their cohort, but none specifically analyzed QoL outcomes in this particular subgroup. Based on findings in the age-heterogeneous population (age range 20–100 years), QoL seems to be correlated with the type of diagnosed carcinoma, length of stay, depression and severe symptom burden.

**Conclusion:**

We were unable to find any research directly exploring QoL and its determinants among cancer patients 80 years and older since none of the included studies presented specific analysis of data in this particular age subgroup. This finding represents a major gap in the knowledge base in this patient group. Based on this finding, we strongly recommend future studies that include this increasingly important and challenging patient group to use valid age- and diagnosis-specific QoL instruments.

## Introduction

The number of people aged 80 years and older is expected to increase in Europe [1], and with advancing age the risk of being diagnosed with cancer increases: 36% of all men and 29% of all women older than 75 years are currently diagnosed with cancer [2]. An increase in the total number of cancer cases among people 80 years and older means that more in-hospital cancer treatment will be required. In 2018, 25,444 (11,4%) people aged 80 years and older were hospitalized because of cancer in Norway [2]. The main causes of hospital admission were cancer progression, cancer-related signs and symptoms (febrile neutropenia, infection, pain, fever and dyspnea), treatment-related complications and end-of-life support [3, 4].

Cancer treatment can be both complex and difficult for patients aged 80 years and older, because of health and comorbidities [5, 6].

The World Health Organization (WHO) defines quality of life (QoL) as “an individual’s perception of their position in life, in the context of the culture in which they live and in relation to their goals, expectations, standards and concerns” [7]. Currently, the term QoL is often used interchangeably with health-related QoL (HRQoL) and is measured by the scores of either a generic or disease-specific QoL questionnaire [8]. Generic means a general questionnaire regardless of the illness or condition of the patient, whereas disease-specific instruments focus on the issues of particular concern to patients with the disease [9, 10]. Generic and disease-specific questionnaires are generally accepted as multidimensional assessments of how disease and treatment affect a patient’s sense of overall functioning and well-being [11]. Padilla et al. [12] defined HRQoL as “a personal, evaluative statement summarizing the positivity or negativity of attributes that characterize one’s psychological, physical and social functioning, and spiritual well-being at a point in time when health, illness, and treatment conditions are relevant” (p 301–308). Most QoL instruments developed over the past 10 years reflect elements of the approach advocated by Padilla et al. [12]. This systematic review uses the term QoL, thus indicating a relatively broad definition of QoL. Since there is no clarity about the term QoL and different instruments are used to measure QoL for older cancer patients [10], this systematic review is therefore indicated to provide clarity in this particular area, and identify future research endeavors.

Chronic disease-related symptoms such as fatigue [13], vomiting, nausea, anxiety, depression [14] and pain [5, 15] are common among older cancer patients and further challenge care management. The presence of multiple symptoms and comorbidity was found to be associated with decreased functional status and QoL in cancer patients [13, 14, 16, 17].

A recent systematic review of studies conducted with hospitalized patients undergoing active treatment for cancer or receiving palliative care found that older patients had more adverse health-related outcomes, including more functional dependence, mental distress, and depression, longer hospital stays and higher mortality than younger patients [4]. The presence of multiple symptoms and comorbidity were shown to be associated with decreased functional status and quality of life [13, 14, 16, 17].

Given the multiple symptoms and other challenges such as multimorbidity, polymedication [18, 19] and reduced tolerance for treatment among people aged 80 years and older with cancer, it could be argued that the experience of being hospitalized combined with treatment will adversely affect these patients’ QoL. Accurate diagnosis and appropriate care and treatment of cancer-related problems can improve patients’ QoL [3]. Nevertheless, existing models of health care do not currently meet the needs and expectations of this group of patients very well [20].

To the best of our knowledge, a systematic review exploring QoL among cancer inpatients 80 years and older has not yet been published. We found only one systematic review of QoL during and after cancer therapy among patients 65 years and older, but that study only included patients with colon cancer [5], thus limiting the generalizability to patients with others types of cancer. Individual studies have reported QoL and its determinants by cancer diagnosis in homogeneous groups of older patients, but to date no attempt has been made to systematically evaluate or compare findings across studies among cancer inpatients 80 years and older. This knowledge is essential for facilitating the best possible treatment and care for the elderly cancer patients in hospital. In this systematic review, we explored the following research question:

In studies that included cancer inpatients undergoing treatment aged 80 years and older, how was quality of life measured and reported in this specific subgroup?

## Methods

This review is registered on PROSPERO [21] and was conducted using the Preferred Reporting Items for Systematic Reviews and Analysis (PRISMA) checklist [22] to ensure rigor in conducting and reporting.

### Eligibility criteria

Eligible studies were those published and which examined QoL among patients aged 80 years and older who were undergoing cancer treatment. Cancer treatment was defined as surgery, chemotherapy and radiation therapy. Studies that covered heterogeneous age groups were included if the study included participants 80 years and older. Eligible studies used any design. Eligible studies were those that were available in English, French, German or Spanish. We did not include gray literature, unpublished studies, ongoing clinical trials, theses or dissertations. Studies with low quality were ineligible for inclusion in the systematic review.

### Search strategy

**Fig. 1:**
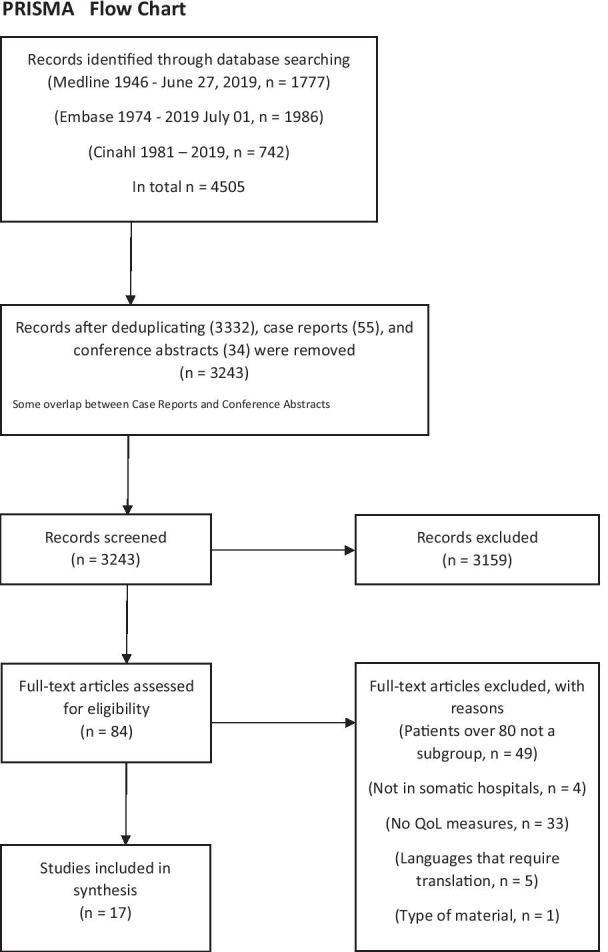
PRISMA flow chart

The following electronic bibliographic sources were searched: Medline (OvidSP 1946–present), Embase (OvidSP 1974–present), and Cinahl (Ebscohost 1981–present). The search was completed in July 2019. The search terms included cancer, hospitalization, elderly patients and quality of life (Fig. 1). Our PICO (population, intervention, comparison and outcome) parameters included cancer patients aged 80 years and older as our population. Our outcome was measures of QoL. The search was created to capture all studies investigating quality of life of older cancer inpatients undergoing cancer treatment. The search strategy was based on the search filter created by Semple et al. [8]. Figure 1 shows the complete search history in a PRISMA flow chart.

### Study selection

We screened all search results by title and abstract using Rayyan software [23]. Two reviewers independently determined the eligibility of all articles by reading the titles and abstracts. The reviewers resolved any disagreement on inclusion or exclusion through discussion. A third reviewer was available if the disagreement could not be resolved. Further, if the title and abstract did not contain enough information to assess eligibility, we screened the full text of the article. Then, in pairs of reviewers (JD & RS and EGB & LE), we screened the full text of each eligible article.

### Data extraction

We developed a data extraction form that enabled us to extract the following data: (1) the author, year and country of publication; (2) the aim or objective of the study; (3) the design and setting; (4) the participants and control group; (5) the QoL instrument used in the study; and (6) the primary results of the study and the authors’ conclusions. The reviewers (EGB, JD, LSPE and RMS) independently extracted the data and double-checked each other’s data. The reviewers resolved any disagreement about data extraction by discussion.

### Quality appraisal

We assessed the quality of the 17 included articles by using the Critical Appraisal Skills Programme (CASP), which comprises checklists adapted to the various study designs. The reviewers independently evaluated each article and resolved disagreements by consensus. Critical assessment of the studies was graded according to different design-specific CASP checklists, from 25% of criteria met, 50% of criteria met, 75% of criteria met to 100% of criteria met CASP checklists [24, 25]. The last column of Table 1 reports the result of each study. Studies were appraised as having high quality when 100% of the criteria were met. Studies were classified as having a risk of low quality when 25% of criteria were met and were excluded from the systematic review. The main methodological drawbacks were reported.

**Table 1:** Results and characteristics of the included studies

Nr	Author, country	Aim	Design and setting	Participants and control group	QoL instruments used	Results	Authors' conclusions	CASP score^a^
				^¤^Number of cancer inpatients older than 80 years of age in the study sample not specified		^◊^Subgroup estimates of QoL in inpatients more than 80 years of age not stated		
1	Alaloul et al., USA [35]	To identify a relationship between patient satisfaction with the hospital experience and HRQoL and to determine the predictors of each variable in cancer survivors	A descriptive, cross-sectional design Two acute cancer care units	50 patients with cancer in two adult oncology units in an academic health sciences center Age range 18–80 years or older (two patients 80 years or older)	QOL-CS (cancer survivor)	Patients with public insurance, diagnosed for 6–10 years and diagnosed for 11 years or longer had lower QoL-CS scores. Patient demographics were related to patient satisfaction and QoL^◊^	Physical well- being, social well- being and time since cancer was related to patient satisfaction and QoL	***
2	Leak Bryant et al., USA [34]	Examine symptoms, mobility and function and QoL in adults with acute leukemia	7-day prospective study Hospital	49 patients with a mean age of 51.6 years (SD 15.8, range 21–88)^¤^	FACT-Leuk, v. 5.0 PROMIS Global Health	Global mental health and pain intensity did not change significantly. Global physical health significantly improved. Fatigue, anxiety, depression and sleep disturbance decreased significantly. QoL increased significantly. Median LOS 33.2 days (SD 10, range 12–63)^◊^	The significant decrease in anxiety and fatigue during hospitalization may be attributable to understanding of the disease process, familiarity with the staff and ability to communicate concerns	****
3	El-Jawahri et al., USA [24]	To assess and compare the QoL, fatigue and mood of older patients with acute myelogenous leukemia (AML) receiving intensive and non-intensive chemotherapy	Prospective longitudinal cohort study Hospital	100 patients > 60 years with a new diagnosis of AML, median age 71 years (range 60–100)^¤^	FACT-Leuk FACT- Fatigue	Older patients with AML experience improvements in their QoL and anxiety while undergoing treatment. Patients receiving intensive and non-intensive chemotherapy have similar QoL and mood trajectories^◊^	The lived experience of older patients receiving intensive chemotherapy was similar to those receiving non-intensive therapy with respect to QoL, fatigue and symptoms of depression and anxiety	****
4	Gu et al., China [32]	To examine the prevalence and correlates of depression and its impact on HRQoL in lung cancer patients	Prevalence study Hospital	148 patients, mean age 64.8 years (SD 11.5, range 20–99)^¤^	WHOQOL- BREF	The prevalence of depression was 43%: men, 39%, women, 50%. Depressed patients had significantly poorer HRQoL than non-depressed patients in terms of all four domains; physical (P < 0.001), psychological (P < 0.001), social (P < 0.001) and environmental ( P < 0.001)^◊^	Depression is prevalent in inpatients with lung cancer and independently associated with poor HRQoL	***
5	Holloway, USA [25]	Assess the association between preoperative QoL and postoperative LOS in colorectal cancer patients after surgical therapy	Prospective cohort study, 1999–2002 Hospital	70 patients Median age 65 years (range 51 -85)^¤^	FACT-C	Poorer pretreatment FACT-C scores (95% CI 1.1–15.6) were significantly associated with increased LOS. Median LOS for the entire group was 6 days (range 3–25). Pretreatment HRQoL scores as measured by FACT-C may benefit in predicting LOS^◊^	Such information may be an important and currently neglected means of risk-adjusting populations undergoing surgery for colorectal cancer for this outcome	***
6	Ishihara, Japan [31]	Evaluate the long-term QoL of patients who underwent total gastrectomy for cancer	Prospective, cross-sectional survey Hospital	51 patients with stomatic cancer, TNM stages I, II or III a Age range 39–82 years^¤^	EORTC-QOL- C30	Comprehensive QOL was good in 20, slightly poor in 17 and poor in 12 (41%) of the 29 patients with good ADL. Dumping symptoms developed in 13 patients (26%), 2 of whom had severe condition. Clear decreases in physical and mental strength (spiritual energy) were reported by 10 and 8 patients^◊^	It is important to evaluate surgical results also with regard to the patients' long-term postoperative QoL	**
7	Jasinska et al., Poland [27]	Assessment of change of QoL in hospitalized terminally ill palliative cancer patients	Prospective cohort 2007–2009 [25] Hospital	41 inpatients, mean age 68 years (range 46–85)^¤^	EORTC QLQ- C15—PAL	Overall QoL was in correlation with the type of diagnosed carcinoma. During the end-of-life care performed in the palliative care unit, the subjective QoL and emotional functioning in patients did not worsen, and in some patients the above parameters improved^◊^	The effectiveness of palliative care relating to overall QoL did not differ significantly among patients with various types of tumors	***
8	Jia et al., China [20]	Investigate cancer-related depression and the relationship between symptoms of depression and QoL	Prevalence study Hospital	262 inpatients with cancer of the digestive system, 50 pancreatic, 60 liver, 50 esophageal, 50 gastric and 52 colorectal cancer patients Four age strata from 20 to 85 years¤	EORTC-QLQ- C30 EORTC-QLQ-PAN-26	The incidence of depression among pancreatic cancer patients was significantly higher than among other types of digestive cancer. Compared with other groups with depression, the QoL of pancreatic cancer patients in each functioning scale was significantly worse, while the symptoms of fatigue and pain were significantly more severe◊	Depression significantly lowers QoL in pancreatic cancer patients	****
9	Jocham et al., Netherlands [39]	QoL assessment of terminally ill patients with cancer, the changes in time, and differences between the groups	Prospective cohort and longitudinal (7 days) 10 palliative home care services, one palliative care unit in a general hospital	Stratified random sample of 121 cancer@@patients, 64 inpatients, mean age 64.4 years (range 35–94) 57 home care patients, mean age 61,7 years¤	EORTC- QLQ C30	The hospital group showed a statistically significant and clinically relevant decrease in nausea and vomiting, pain and dyspnea. The home care group had statistically significant improvements in the domains of QoL function: cognitive, physical, role, emotional, social◊	EORTC QLQ-C30 can be a useful measure for the QoL of German cancer patients under palliative care symptom control and were sensitive to changes over time	***
10	Nafteux et al., Belgium [28]	Identify preoperative QoL factors predicting prolonged hospital stay after esophagectomy caused by cancer	12 months prospective cohort Hospital	455 at baseline 330 after 2 years Mean age 63 (range 34–88)¤	EORTC-QLQ- C30 EORTC-QLQ-OES-18	Low QoL predicts LOS. Prognostic factors, LOS (> 10 days): medical HR, 6.2 (3.62–10.56, surgical HR 2.79 (1.70 -4.59), morbidity, readmittance to intensive care unit HR 33.82 (4.55–251.21); poor physical functioning HR 1.89 (1.14–3.14)◊	Better perception of preoperative physical functioning might have a beneficial effect on LOS. Early discharge correlates with improved postoperative HRQoL outcomes	****
11	Peters & Sellick, Australia [36]	To report symptom experience, physical, mental health, perceived control of the effects of cancer and QoL and the predictors of QoL of terminally ill cancer patients	Comparative cohort study Hospital and home-based	32 inpatients and 26 home-based, mean age 67.8 years (range 40–92)¤	EORTC QLQ- C30 [4]	Patients receiving home-based services had statistically significantly less symptom severity and distress, lower depression scores and better physical health and QoL than those receiving inpatient care. Better global physical health, greater control over the effects of cancer and lower depression scores were statistically significant predictors of higher QoL◊	Early detection and management of physical and mental symptoms and strategies that will empower patients to have a greater sense of control over their illness and treatment may impact QoL	****
12	Shinozaki et al., Japan [40]	Investigate QoL and functional status of terminally ill head and neck cancer patients	Multicenter, prospective, observational study Hospital	100 patients, 72 were observed until death Median age 69 years (range 37–94)¤	EORTC QLQ- C15-PAL	No significant difference in QoL score between baseline and week 3. The route of nutritional intake (nasogastric tube versus percutaneous gastric tube) predicted the length of hospital stay (64 versus 21 days, P = 0.04) and may play a role for QoL◊	Feeding tube type could have the most impact on QoL	****
13	Stromgren et al., Denmark [29]	To study disease-related and treatment-related HRQoL, functional capacity and symptoms of patients with cancer	5-week prospective cross-sectional survey Hospital	124 patients, mean age 59 years (range 21–88)¤	EORTC QLQ C30 WHO PS	HRQoL, role and social functioning were more severely impaired in hematology patients than in cancer patients, whereas pain and constipation were worse for cancer patients than for hematology patients◊	Patients (hematology and cancer) had pronounced symptoms and low QoL	****
14	Torvik et al., Norway [30]	Examine differences in pain, pain management, satisfaction with pain management, QoL and pain interference between middle-aged and older patients with bone metastases	Prevalence study Hospital	79 patients, 39 = > 65 years, Mean age 76, Range (66—88)¤	Global QoL (single item)	Older patients, higher scores for "worst pain" (P = 0.04), "pain severity intensity" (P = 0.03) but received strong opioids for their cancer pain significantly less often than middle-aged patients (P = 0.02). Linear association between increasing age and decreasing pain (P = 0.002)◊	These results indicate that more focus is needed on pain management and QoL in older cancer patients with bone metastases	***
15	Van der Walde et al., USA [33]	Investigate geriatric assessment as a predictor of tolerance, QoL and outcomes in older patients with head and neck cancer and lung cancer receiving radiation therapy	Prospective cohort study Hospital	46 patients, mean age 72.5 Range (6592) 13% of sample was aged 80 + [6]¤	EORTC QLQ- C30	Patients with 1-ADL dysfunction at baseline were more likely to have reduced HRQoL on role and social function after radiation therapy. Patients with dysfunction had lower baseline HRQoL scores◊	Pretreatment dysfunction was associated with continued decline and lack of recovery of HRQoL in this patient population	****
16	Wittmann-Vieira & Goldim, Brazil [37]	Evaluate the decision-making process and QoL of adult cancer patients	Cross-sectional Inpatients in a palliative unit	89 patients, mean age 53 years, range (25—85)¤	WHOQOL- OLD WHOQOL- BREF	In the domains of WHOQOL-OLD, the social participation had the lowest mean and intimacy had the best. In terms of application of the domains of the WHOQOL-BREF instrument, the physical domain had the lowest mean, while the environment had the best performance◊	Patients demonstrated satisfaction with the capacity to establish social relationships, personal and intimate, when answering WHOQOL-OLD and WHOQOL-BREF, even while hospitalized	***
17	Lee et al., South Korea. [38]	Explore the QoL and performance status as prognostic indicators of survival	Retrospective cohort study Inpatients in palliative care	162 inpatients with advanced cancer, age range 40–86 years¤	EORTC-QLQ- C15 PAL ECOG	Physician-reported PPS significantly predicted survival (HR 0.493; P < 0.001). From the EORTC QLQ-C15-PAL, patient-reported physical functioning predicted survival (HR = 0.65; P < 0.001). the other six domains of EORTC QLQ-C15-PAL (global health status, emotional functioning, fatigue, nausea and vomiting, appetite loss and constipation) were significantly related to survival after adjustment◊	QLQ is useful even for patients in their final month of life. Cancer anorexia-cachexia syndrome-related symptoms may be independent prognostic symptoms	***

^a^ Critical assessment of the studies was graded according to different design specific CASP checklists: *25% of criteria met; **50% of criteria met; ***75% of criteria met; ****100% of criteria met [26]

^¤^Number of cancer inpatients older than 80 years of age in study sample not specified

^◊^Subgroup estimates of QoL in inpatients older than 80 years of age not stated.

ADL: activities of daily living; EORTC: European Organization for Research and Treatment of Cancer; FACT-Leuk: Functional Assessment of Cancer Therapy- Leukemia; FACT-C: Functional Assessment of Cancer Therapy - colorectal cancer; FACT-Fatigue: Functional Assessment of Cancer Therapy - fatigue; HRQoL: health-related quality of life LOS: length of stay; OES-18: Oesophageal cancer module, 18 items; PPS: Palliative Performance Scale; PROMIS: Patient-Reported Outcomes Measurement Information System; QoL: quality of life; QLQ-C15-PAL: Quality of Life Questionnaire Core 15 for Palliative Care; QlQ-C30-PAL: Quality of Life Questionnaire Core 30 for Palliative Care; QOL-CS: Quality of Life Patient Cancer Survivor version; QLQ-PAN-26: Quality of Life Questionnaire Core 26 for Pancreatic Cancer; WHOQOL-BREF: World Health Organization Quality of Life Questionnaire, brief version; WHOQOL-OLD: World Health Organization Quality of Life Questionnaire in old people; WHO PS: World Health Organization Performance Scale

### Data synthesis and analysis

The included studies differed in study design, cancer population, QoL instruments and statistical analysis used, and the results were therefore synthesized narratively (Table 1).

## Results

We included 17 studies that had patients aged 80 years and older in their cohorts. Since none of the studies presented estimates of QoL in this particular subgroup of patients, no direct evidence was found on QoL among cancer inpatients aged 80 years and older. Only one of the included studies used an age-specific QoL instrument.

### Study selection

We pooled the search results from the three databases. The review group screened 2953 titles and abstracts according to a predefined set of inclusion criteria, found 84 eligible studies, screened them in full text and excluded 67 of these. The primary reason for exclusion at this stage was the fact that patients 80 years and older were not included or that QoL was not measured (Fig. 1).

### Characteristics of the included studies

This review included 2005 participants from 11 countries. Most of the studies were conducted in the United States (*n* = 5), followed by China and Japan (*n* = 2). In studies that provided information on participants’ ages, the ages ranged from 20 [19] to 100 years [24], and only two studies stated how many participants were older than 80 years. No studies analyzed QoL outcomes in the subgroup of patients aged 80 years and older. The duration of the study periods ranged from 7 days to 31 months. The sample sizes ranged from 32 to 455. The studies used retrospective (*n* = 1) and prospective (*n* = 10) cohorts and cross-sectional (*n* = 6) designs. Twelve of the studies included only hospitalized patients [20, 24, 25, 27–35], and one study included both hospital- and home-based patients [36]. Two studies included inpatients in the palliative care unit in the hospital who were undergoing cancer treatment [37, 38], and one study included patients from palliative home care services and the palliative unit of a hospital [39]. One study [40] was a multicenter study from 11 cancer centers and a university hospital (Table).

### Instruments used to measure QoL

QoL instruments are categorized as either disease-specific or generic or overall [41]. Most (*n* = 11) of the 17 studies used disease-specific instruments to measure QoL in cancer [20, 25, 27–29, 31, 33, 36, 38–40]. The disease-specific instruments included European Organization for Research and Treatment (EORTC) Quality of Life Questionnaire Core 30 (EORTC-QLQ-C30) [20, 28, 29, 33, 36, 39, 40], EORTC Quality of Life Care Palliative 15 (EORTC QLO-C15 PAL) [27, 38, 40], EORTC Quality of Life esophagus cancer version (EORTC QLQC30-OES-18) [28] and EORTC Quality of Life pancreas version (EORTC-QLQ-PAN- 26) [20]. In addition to the EORTC QoL instruments, some studies used other disease-specific QoL instruments, including the Functional Assessment of Cancer Therapy – Leukemia (FACT-Leuk) [24, 34] and the Functional Assessment of Cancer Therapy – Colorectal Cancer (FACT-C) instruments [20, 24] and the Functional Assessment of Cancer Therapy Fatigue (FACT-Fatigue) [24] and Quality of Life Patient/Cancer Survivor (QoL-CS) version.

Three of the 17 studies used generic instruments [32, 34, 37]. The generic QoL instruments were WHOQOL-BREF [32, 37], PROMIS Global Health [34] and WHOQOL-OLD [37], which has been validated for use with older people. One study used a generic QoL instrument comprising the single item “Overall, how would you presently rate your own life?” [30].

None of the included studies reported on patients’ spiritual QoL domain, regardless of the study sample’s age.

### How quality of life was reported in the subpopulation of cancer patients older than 80 years

QoL was not estimated among the subgroup of inpatients older than 80 years of age in any of the studies, even though this specific subgroup was represented in all study samples.

### How quality of life was reported in the populations studied (age range 20–100 years)

A study of the incidence of pancreatic cancer–related depression among inpatients with cancer found that the type of carcinoma in the digestive system (pancreatic, liver, esophageal, gastric and colorectal) and symptoms of depression were negatively correlated with QoL, as measured by the EORTC-QLQ-PAN-26 questionnaire [20], QoL domains (global health status, physical functioning and emotional functioning) measured by QLQ-C15-PAL predict survival among 162 inpatients with advanced cancer [38]. In a study of the effectiveness of palliative care during the end of life of cancer inpatients with prostate and lung cancer, found that QoL, as measured by QLQ-C15-PAL, was correlated with the type of diagnosed carcinoma [27]. In that study, patients with lung cancer had lower QoL than patients with colon cancer.

In a study comparing one palliative care unit in a general hospital and 10 palliative home care services, the terminally ill cancer inpatients had a statistically significant and clinically relevant decrease in nausea and vomiting pain and dyspnea compared with the terminally ill cancer patients in home care services [39]. Two studies identified quality of life as higher among patients with lower lengths of hospital stays [26, 28]. Postoperatively, patients (with esophageal and gastroesophageal junction cancer) with length of hospital stays < 10 days had significantly better QoL scores in the functional scales (physical, emotional, social and role functioning) and in symptom scales (fatigue, nausea, dyspnea, appetite loss and dry mouth) at 3 and 12 months compared with patients with a length of stay > 10 days [28]. Lower pretreatment QoL (as measured by FACT-C) was significantly correlated with increased length of stay among inpatients undergoing surgery for colorectal cancer [26].

Terminally ill cancer inpatients in a palliative care center had statistically significantly more symptom severity and distress, higher depression score and worse physical health and QoL than the terminally ill cancer patients receiving home-based services [36].

Low QoL and severe symptom burden, especially fatigue and appetite loss, were observed among the inpatients with malignant disease in one study [29]. Patients with instrumental activities of daily living (IADL) dysfunction at baseline were more likely to have reduced QoL on role and social functioning after radiation therapy compared with patients without IADL dysfunction [33].

Shinozaki et al. [40] found no significant relationship between QoL scores and functional status among terminally ill inpatients with head and neck cancer, and depressed inpatients with lung cancer had significant worse physical, mental, social and environmental QoL than patients who were not depressed [32]. The age groups did not differ statistically significantly in global QoL among inpatients with bone metastasis, as measured by the single-item questionnaire [30].

Further, the symptoms of fatigue, anxiety and sleep disturbance were associated with reduced QoL among adult inpatients with acute leukemia [34], and QoL was reported to be poor among 41% of the 29 inpatients with high scores in activities of daily living who had undergone total gastrectomy for cancer [31]. QoL was not significantly associated with functional status in a study of terminally ill inpatients with head and neck cancer [40]. All these results are based on analyses of age-heterogeneous cancer inpatients 20–100 years of age.

### Methodological quality of the included studies

Overall, all 17 included studies had high to medium methodological quality according to the CASP assessment and met at least 9 of 12 criteria on the checklists (high quality) (Table 1, last column). The main methodological drawbacks of the included studies were related to question 5 on confounding factors. Eight of the 17 studies did not indicate if they had controlled for confounding factors [25, 27–29, 33, 34, 36, 39], creating difficulty in drawing conclusions on the validity of the results. Five of the studies [26, 27, 34, 35, 39] had limitations related to question 8 on the confidence interval estimate of the HRQoL or QoL outcome. Two of the studies did not report receiving approval by an ethics committee [37, 38].

## Discussion

Although the 17 studies included in this systematic review did not report QoL specifically among cancer inpatients aged 80 years and older, they all included participants in this age group. The studies represent different study designs, age and cancer populations, measurement scales and outcomes. The results of this review nevertheless provide some indirect insights that will contribute to improving the understanding of QoL among patients aged 80 years and older admitted to hospital. The results of this review identified that almost all studies (*n* = 10) measured QoL using instruments that do not measure all aspects of QoL (mental, physical, social and spiritual well-being) as defined by Padilla et al. [12].

The spiritual domain would be especially important to measure among cancer inpatients older than 80 years, since they are approaching the final stage of their life and have a severe disease. Nevertheless, this domain was only included in two instruments used: WHOQOL-OLD [37] and the Quality of Life Index [31]. The articles did not present the results of the analysis of the spiritual domain. Further, the EORTC QLQ-C15-PAL, which does not include the spiritual domain, was used in studies of advanced cancer and terminally ill patients [27, 38, 40]. Spiritual well-being is relevant for older patients undergoing cancer treatment and receiving palliative care [42]. QoL measures should therefore be complemented by questionnaires that include spirituality to aid health care providers in better facilitating the patients’ individual needs at the end of life. The finding that QoL among cancer patients aged 80 years and older has not been investigated with validated diagnosis- and age-specific instruments is important because evidence is needed to inform the development of appropriate health care services for this group of patients.

Hospital patients reported worse physical health, QoL and symptom burden than those in home care services [36, 39] this is due to more severe cases being hospitalized. Symptoms among older patients with cancer are often reported measured with highly relevant measurement instruments for cancer patients [3–5, 13]. However, severe symptoms may reduce patients’ overall QoL because of distress [41].

For older patients with cancer, preoperative QoL, as measured with EORTC-QLQ C30 [25] and FACT-C [28], is beneficial in predicting the length of hospital stay for different types of cancer. Holloway et al. [25] reported a significant association between lower FACT-C score and increased length of stay, where Nafteux et al. [28] reported that QoL, especially poor physical functioning, was an independent prognostic factor for longer hospital stay.

Only two of the studies [31, 35] provided information on Classification of Malignant Tumors TNM staging. One would assume that a large tumor size would inversely correlate with QoL scores. In a recent study of patients with renal tumors, preoperative tumor size did not correlate significantly with self-reported QoL. However, the exception was that patients with the largest tumors (> 7.0 cm) reported significantly worse general health and QoL (questions 29 and 30 in the QLQ-C30 questionnaire) [43]. Studies of head and neck cancer patients identified that tumor size does not correlate with self-reported QoL [44]. Nevertheless, these results are in accordance with the study by Vissers et al. [45] that found that, regardless of cancer type, comorbidity explains more of the variance in QoL than tumor size.

Comorbidities are important factors that often characterize patients aged 80 years and older [16, 46], which in turn may affect their QoL [13]. We expected that comorbidities could be a plausible confounding factor for older people, but only a few studies highlighted this issue [27, 38, 39, 47].

Another important finding of this review was that older patients reported higher levels of pain and received significantly fewer opioids for their cancer-related pain than middle-aged patients, but their overall QoL did not differ significantly [30]. However, global QoL scales provide no information on the different dimensions of QoL, and older patients with higher levels of pain may therefore have differed from middle-aged patients with lower levels of pain on specific dimensions of QoL. Since cancer pain reduces the QoL of older patients and impairs their physical functioning, sleep, activities of daily living, life enjoyment and mood [48], the measurement of QoL among older cancer patients needs to be investigated along all the dimensions of QoL to better understand and meet their care needs. In future studies, multidimensional scales such as the EORTC-QLQ-ELD [48] should be used to obtain information on the dimensions of QoL scales among older people.

### Strengths and limitations of the review

Our review included studies reported in several languages, including English, French, German and Spanish. All 17 studies used QoL as primary outcome. Several methodological issues limit the conclusions of this review. All the included studies had different designs, aims and outcomes. Our review included studies that reported a wide age range (20–100 years) and highlighted the fact that none of the studies reported separate statistical analysis for people 80 years and older. This limitation makes it difficult to understand how patients aged 80 years and older experience their QoL during cancer treatment. Older people are expected to rate their QoL differently from younger people, and different domains of QoL, such as the spiritual domain, are more or less important in different age groups.

The prospective studies show great variation in the sample sizes, from 32 [36] to 455 included participants [28]. The heterogeneity of the questionnaires used to assess QoL makes comparing the results difficult. We found that no studies examined QoL in patients with specific cancer diagnoses. Hence, including different cancer diagnoses in one review might even increase the external validity.

For cost reasons and to ensure the quality warranted by a peer-review process, we did not search gray literature, unpublished studies, ongoing clinical trials, theses or dissertations. We did not include studies written in Chinese, Japanese or Russian.

## Conclusions

This review included 17 studies investigating QoL among cancer inpatients 80 years and older. Several QoL instruments were used, and only one study used an age-specific instrument. None of the studies specifically analyzed QoL outcomes among patients 80 years and older. Therefore, no firm conclusion can be drawn regarding the evidence on the QoL of cancer inpatients 80 years and older. This finding represents a major gap in the knowledge base in the cancer literature. Based on this finding, we strongly recommend future studies in this increasingly important and challenging patient group using valid age- and diagnosis-specific instruments and conducting subgroup analysis for patients 80 years and older.

## Data Availability

Not applicable.

## Abbreviations

QoL: quality of life
CASP: critical appraisal skills programme
ADL: activities of daily living
BPI: brief pain inventory
CIPM: clinical index of pain management
ECOG: eastern cooperative oncology group
EORTC: European organisation for research and treatment of cancer
FACT-Leuk: functional assessment of cancer therapy– Leukemia
FACT-C: functional assessment of cancer therapy – colorectal cancer
FACT-Fatigue: functional assessment of cancer therapy – Fatigue
OES-18: esophageal cancer module, 18 items
PROMIS: global health patient reported outcome measure global health
QLQ-C15-PAL: quality of life questionnaire core 15 for palliative care
QlQ-C30-PAL: quality of life questionnaire core 30 for palliative care
QOL-CS: quality of life patient/cancer survivor version
QLQ-PAN- 26: quality of life questionnaire core 26 for pancreatic cancer
WHOQOL-BREF: world health organization quality of life questionnaire – brief version
WHOQOL-OLD: world health organization quality of life questionnaire for older adults
