# Correction to: Quality of life among cancer inpatients 80 years and older: a systematic review

**DOI:** 10.1186/s12955-021-01849-y

**Published:** 2021-09-03

**Authors:** Jorunn Drageset, Reidun Karin Sandvik, Leslie Sofia Pareja Eide, Gunhild Austrheim, Mary Fox, Elisabeth Grov Beisland

**Affiliations:** 1 Faculty of Health and Social Sciences, Western Norway University of Applied Sciences, 5063 Bergen, Norway; 2 Department of Public Health and Primary Health Care, University of Bergen, Bergen, Norway; 3 York University, Durham, NC USA

## Correction to: Health Qual Life Outcomes (2021) 19:98 10.1186/s12955-021-01685-0

The original article [1] contained an error in Affiliation #3 which has since been corrected.
